# Structural snapshots of the kinesin‐2 OSM‐3 along its nucleotide cycle: implications for the ATP hydrolysis mechanism

**DOI:** 10.1002/2211-5463.13101

**Published:** 2021-02-28

**Authors:** Paloma F. Varela, Mélanie Chenon, Christophe Velours, Kristen J. Verhey, Julie Ménétrey, Benoît Gigant

**Affiliations:** ^1^ Université Paris‐Saclay CEA CNRS Institute for Integrative Biology of the Cell (I2BC) Gif‐sur‐Yvette France; ^2^ Department of Cell and Developmental Biology University of Michigan Medical School Ann Arbor MI USA

**Keywords:** ATP hydrolysis mechanism, kinesin, microtubule, motor protein, X‐ray crystallography

## Abstract

Motile kinesins are motor proteins that translocate along microtubules as they hydrolyze ATP. They share a conserved motor domain which harbors both ATPase and microtubule‐binding activities. An ATP hydrolysis mechanism involving two water molecules has been proposed based on the structure of the kinesin‐5 Eg5 bound to an ATP analog. Whether this mechanism is general in the kinesin superfamily remains uncertain. Here, we present structural snapshots of the motor domain of OSM‐3 along its nucleotide cycle. OSM‐3 belongs to the homodimeric kinesin‐2 subfamily and is the *Caenorhabditis elegans* homologue of human KIF17. OSM‐3 bound to ADP or devoid of a nucleotide shows features of ADP‐kinesins with a docked neck linker. When bound to an ATP analog, OSM‐3 adopts a conformation similar to those of several ATP‐like kinesins, either isolated or bound to tubulin. Moreover, the OSM‐3 nucleotide‐binding site is virtually identical to that of ATP‐like Eg5, demonstrating a shared ATPase mechanism. Therefore, our data extend to kinesin‐2 the two‐water ATP hydrolysis mechanism and further suggest that it is universal within the kinesin superfamily.

**Protein Database entries:**

7A3Z, 7A40, 7A5E.

## Graphical abstract

Here, we report the crystal structure of the *Caenorhabditis elegans* kinesin‐2 OSM‐3 motor domain in three nucleotide states. We have in particular captured OSM‐3 bound to an ATP analog in a microtubule‐bound ATP‐kinesin conformation, ready to hydrolyze ATP. These data suggest that the ATPase mechanism is conserved within the kinesin superfamily.

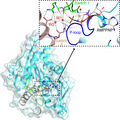

**Abbreviation**
SEC‐MALLS: size‐exclusion chromatography coupled to multi‐angle laser light scattering

Motile kinesins are transport proteins powered by ATP hydrolysis and involved in intracellular trafficking, carrying loads as they translocate along the microtubule tracks [1]. Kinesins share a conserved motor domain that contains the binding sites for ATP and for microtubules. The motor domain is however not fully conserved, and based on sequence similarities, most kinesins can be assigned to one of the fourteen to eighteen families that have been proposed based on phylogenetic analysis [3, 4, 5, 6].

Structures of the motor domain of members from most kinesin families have been determined [7]. These structures have illuminated how subdomains of the motor domain reorganize along the kinesin mechanochemical cycle [8, 9] and defined three main structural states related to the three principal nucleotide states of kinesin: the ADP state, generally characterized by a low affinity for microtubules, and the nucleotide‐free and ATP states, both of high affinity for microtubules [10]. These subdomain movements are coupled to major structural rearrangements of phosphate‐sensing loops, named Switch 1 and Switch 2 (see Fig. 1A for secondary structure nomenclature).

**Fig. 1:**
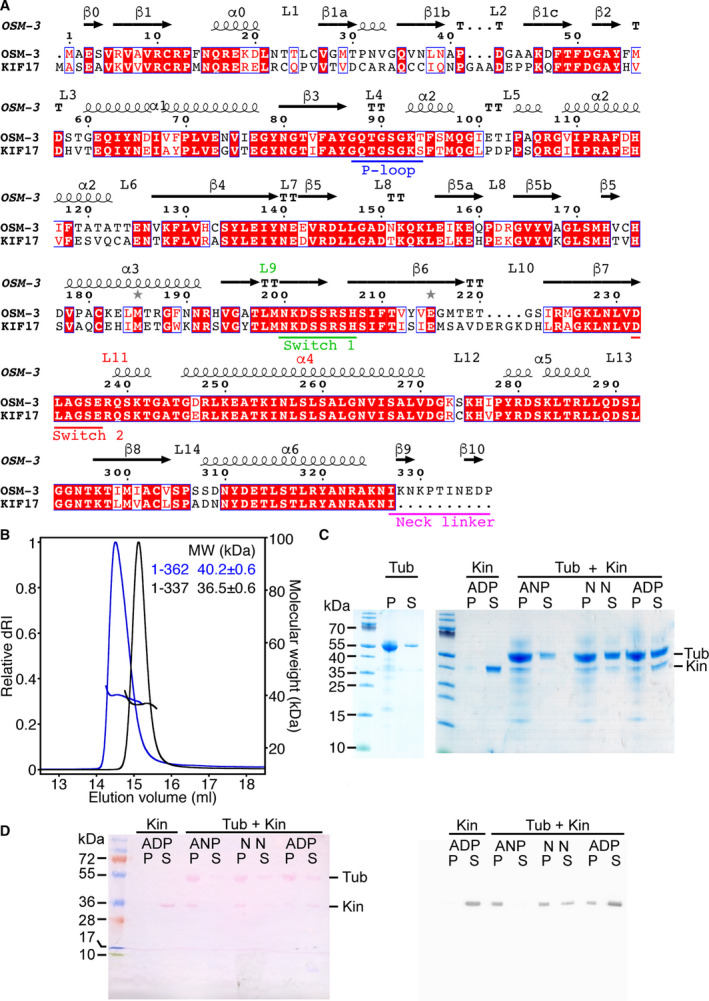
Biochemical characterization of OSM‐3. (A) ENDscript [55] output of the sequence alignment of the motor domains of human KIF17 (NCBI reference NP_065867.2) and of *Caenorhabditis elegans* OSM‐3 (NP_001367796.1). Identical residues are colored in white on a red background; similar residues are in red. The ATP‐binding motifs (P‐loop, Switch 1 and Switch 2) are underlined in blue, green and red, respectively. The secondary structure elements are derived from the crystal structure of AMPPNP‐OSM‐3 and are labeled according to established nomenclature. The OSM‐3 sequence includes the neck linker residues that are ordered in the structures we determined. (B) SEC‐MALLS analysis of the 1–337 and 1–362 OSM‐3 constructs (black and blue curves, respectively). The differential refractive index (normalized dRI, left axis) and molecular mass (right axis) are plotted as a function of the column elution volume. (C, D) Interaction with microtubules of the OSM‐3 1–362 construct (panel C) and 1–337 construct (panel D). OSM‐3 (Kin) was incubated with tubulin (Tub) under microtubule‐assembly conditions, in the presence of 1 mm AMPPNP (ANP) or 2 mm ADP, or without added nucleotide (N N, no nucleotide). In panel C, after ultracentrifugation, the equivalent of 0.8 µg OSM‐3 and 6 µg tubulin [supernatant (S) + pellet (P)] was submitted to SDS/PAGE analysis. In panel D, the equivalent of 1 µg OSM‐3 and 7.5 µg tubulin was submitted to SDS/PAGE followed by western blot analysis (Left, ponceau staining; Right, anti‐His chemiluminescent detection).

These data have also provided insight into the mechanism of ATP hydrolysis, which is enhanced upon kinesin binding to microtubules [11]. A mechanism involving two water molecules, a lytic one and a relay one, has been inferred from the structure of the isolated kinesin‐5 Eg5 motor domain bound to the ATP analog AMPPNP [12]. Support for the catalytic two‐water mechanism was provided when the same conformation, with the nucleotide‐binding site in a ‘closed’ state, was found for the isolated kinesin‐4 KIF4 motor domain in complex with AMPPNP [13]. This ‘closed’ conformation is characterized in particular by the Switch 1 region adopting an extended hairpin structure and the Switch 2 region being fully ordered. Such a conformation is also adopted by kinesin‐1 [14] and kinesin‐13 [15, 16, 17] motor domains bound to an ATP analog and in complex with tubulin. This conformation is further compatible with the cryo‐EM map of several kinesin‐decorated microtubules, including the kinesin‐6 MKLP2 [18] and the kinesin‐8 KIF18A [19]. Thus, high‐resolution structures of the isolated motor domains of ATP‐like kinesin‐4 and kinesin‐5 display microtubule‐bound conformations observed in lower resolution structures where the water molecules cannot be explicitly identified. Taken together, these data suggest that the ATPase mechanism is conserved across kinesin families. However, in structures of other isolated kinesin motor domains bound to AMPPNP (e.g., kinesins of classes 3 and 10), neither the Switch 1 region nor that of Switch 2 were entirely ordered [20, 21, 22]. Therefore, the ‘closed’ conformation of the nucleotide‐binding site has not been observed in these kinesins, raising the possibility of a different ATP hydrolysis mechanism.

We report here the structural characterization of OSM‐3, a kinesin‐2 from *Caenorhabditis elegans*. The kinesin‐2 family is subdivided into two subgroups [23]. One of them is composed of heterotrimeric proteins made of two distinct kinesin chains and a non‐motor‐associated protein. OSM‐3 and its human homologue KIF17 belong to the second subfamily, for which no structure is available, and are homodimeric proteins. Similar to KIF17, OSM‐3 is involved in intraflagellar transport in the distal part of the cilium [24], and both proteins are autoinhibited at the resting state [25, 26]. We have determined the structure of the motor domain of OSM‐3 either bound to ADP or to AMPPNP, or devoid of a nucleotide. These structural snapshots of OSM‐3 along its nucleotide cycle further support a two‐water ATP hydrolysis mechanism similar to that of the kinesin‐5 Eg5 and kinesin‐4 KIF4 motors.

## Results and Discussion

### Biochemical characterization of the OSM‐3 motor domain

In our efforts to characterize the structural mechanism of KIF17‐related kinesins, we first tried to produce different motor domain constructs of vertebrate KIF17 but the proteins were insoluble in our hands when expressed in bacteria. We thus switched to the *C. elegans* homologue OSM‐3, whose motor domain shares 69% sequence identity (82% sequence similarity) over 327 residues with that of human KIF17 (Fig. 1A). We produced and purified an OSM‐3 construct (aa. 1–362) which comprises the core motor domain (through the α6 helix) and 35 additional residues C‐terminal to the motor domain. To enhance the likelihood to obtain crystals, we also produced a shorter construct (aa. 1–337), formed by the core motor domain and 10 residues C‐terminal to it. Therefore, these two constructs contain all or part of the neck linker, a 14–18 residue peptide C‐terminal to the motor domain which is a key element for transmission of movement in kinesins [27, 28, 29, 30]. Size‐exclusion chromatography coupled to multi‐angle laser light‐scattering (SEC‐MALLS) analysis indicated that both OSM‐3 constructs were monomeric in solution (Fig. 1B), with estimated masses of 36.5 ± 0.6 kDa (1–337 construct) and 40.2 ± 0.6 kDa (1–362 construct), close to the mass predicted from their sequence (38.2 and 41.1 kDa, respectively). OSM‐3 constructs also interacted with microtubules in the presence of AMPPNP and in the absence of nucleotide, but less in the presence of excess ADP (Fig. 1C,D). To gain further insights into the structural mechanism of OSM‐3, both constructs were then submitted to crystallization experiments and the structure of OSM‐3 in different nucleotide states was determined.

### The ADP‐OSM‐3 structure

Several crystal forms were obtained from both OSM‐3 constructs stored in an ATP‐based buffer. The first structure was determined by molecular replacement taking that of the motor domain of the human heterodimeric kinesin‐2 KIF3B (PDB code 3B6U [31]) as a search model. This first OSM‐3 structure then served as a model for the determination of the structure of the other crystal forms. The OSM‐3 conformation was mostly similar in these different crystal forms, whatever the OSM‐3 constructs, with rmsd ranging from 0.5 to 0.8 Å. The main differences concern variations in the α0 helix and in loop L5, most likely induced by different crystal packing contacts; these variations also reflect a mobility of these elements with respect to the motor domain core. The best crystal diffracted X‐rays up to 2.1 Å resolution (Table 1) and was used in the analysis presented below.

**Table 1:** Data collection and refinement statistics. n.a., not applicable

	ADP‐OSM‐3	Nucleotide‐free OSM‐3	AMPPNP‐OSM‐3
**Data collection** ^a^			
Space group	C2	P2_1_	C222_1_
Cell dimensions			
*a*, *b*, *c* (Å)	136.9, 62.1, 45.0	42.8, 155.2, 63.7	100.0, 102.2, 140.9
α, β, γ (°)	90.0, 90.2, 90.0	90.0, 109.0, 90.0	90.0, 90.0, 90.0
Resolution (Å)	45–2.09 (2.15–2.09)	47.6–2.30 (2.36–2.30)	48–1.90 (1.95–1.90)
*R*_meas_	0.188 (2.25)	0.121 (1.83)	0.098 (3.56)
*I*/σ*I*	7.5 (0.8)	8.6 (1.0)	13.7 (0.8)
CC_1/2_	0.994 (0.394)	0.997 (0.625)	0.999 (0.397)
Completeness (%)	99.4 (95.0)	99.6 (95.4)	99.8 (94.6)
Multiplicity	6.9 (6.6)	7.0 (6.6)	13.6 (13.2)
**Refinement**			
Resolution (Å)	45–2.09	47.6–2.30	48–1.90
No. of reflections	22 197	34 801	56 649
*R*_work_/*R*_free_	0.211/0.247	0.225/0.254	0.206/0.231
No. Mol/ua No. atoms	1	2	2
Protein	2308	4545	5160
Ligands	28	22	126
Solvent	141	65	218
*B* factors			
Protein	52.7	87.2	60.8
Ligands (nucleotide + Mg^2+^)	41.3 (41.3)	73.5 (n.a.)	77.4 (57.2)
Solvent	50.6	64.3	56.9
Coordinate error (Å)	0.32	0.40	0.30
R.m.s.d.			
Bond lengths (Å)	0.008	0.008	0.008
Bond angles (°)	0.95	1.02	0.95
Ramachandran (%)			
Favored region	99.3	96.2	98.8
Allowed region	0.7	3.5	1.2
Outliers	0	0.3	0
PDB id code	7A3Z	7A40	7A5E

^a^ Data were collected on a single crystal. Values in parentheses are for the highest‐resolution shell.

**Fig. 2:**
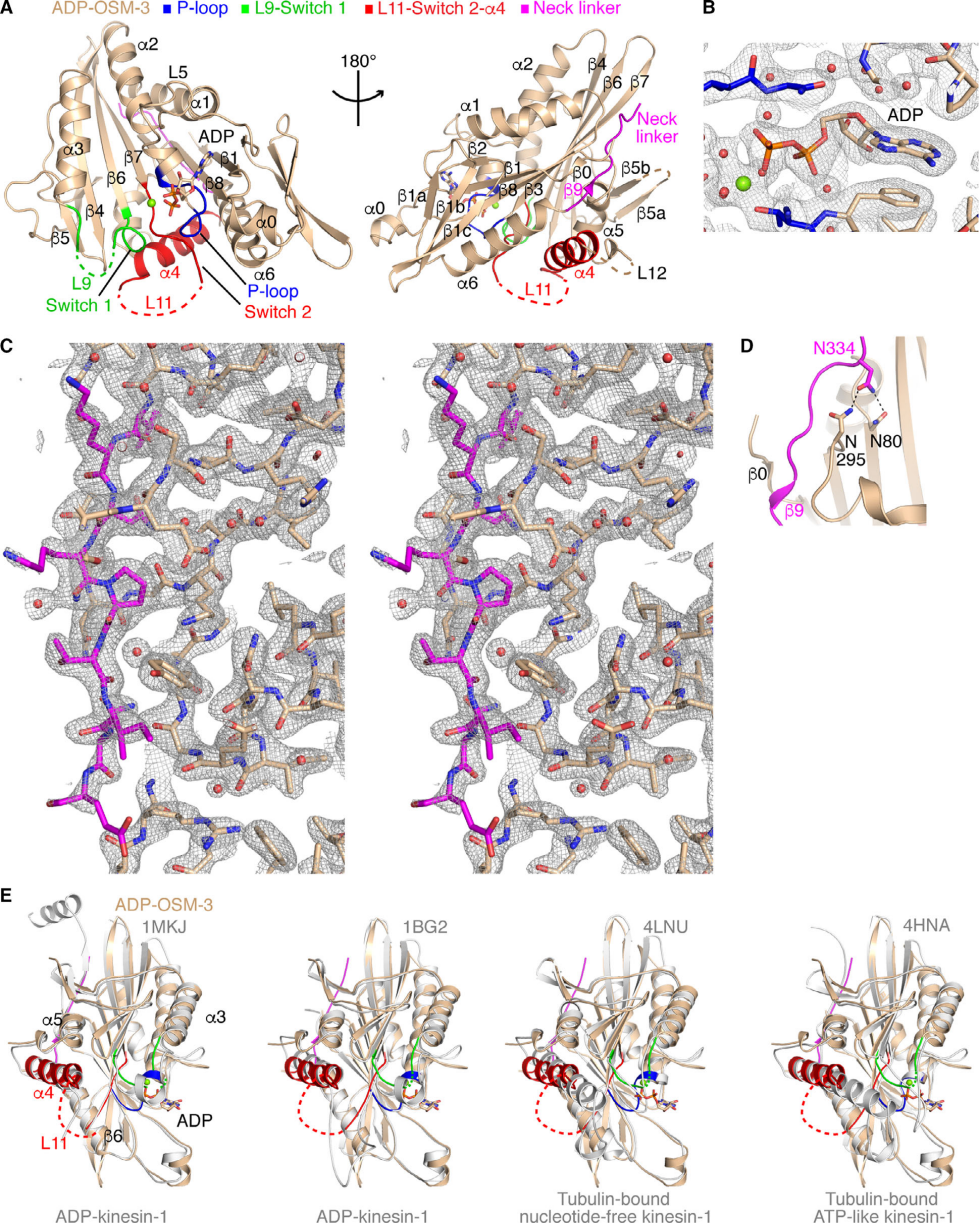
The structure of ADP‐OSM‐3 (1–362 construct). ADP‐OSM‐3 is in wheat, except for the P‐loop in blue, the L9‐Switch 1 region in green, the Switch 2‐L11‐α4 region in red, and the C‐terminal neck linker peptide in magenta. (A) Two overviews of ADP‐OSM‐3. The Mg^2+^ ion is shown as a green sphere. Loops that are not defined in the electron density maps are shown as dashed lines. (B) The 2 *F*_obs_ − *F*_calc_ electron density map (contoured at the 1 σ level) of ADP‐OSM‐3 centered on ADP. (C) Stereo view of the 2 *F*_obs_ − F_calc_ electron density map (contoured at the 1 σ level) of ADP‐OSM‐3 centered on the neck linker. Neck linker residues, from Ile327 (Top) to Glu335 (Bottom), are in magenta. (D) Stabilization of the neck linker by the cover neck bundle and the Asn latch. (E) Superposition of kinesin‐1 structures (gray) with that of ADP‐OSM‐3. From left to right: comparison with ADP‐kinesin‐1 with a docked neck linker (PBD id 1MKJ) or with a disordered neck linker (1BG2), with tubulin‐bound nucleotide‐free kinesin‐1 (4LNU), and with tubulin‐bound ATP‐like kinesin‐1 (4HNA). Only the nucleotide and the Mg^2+^ ion from ADP‐OSM‐3 are shown.

The OSM‐3 motor domain shows the typical kinesin fold [32], with a central eight‐stranded β‐sheet surrounded by 3 α‐helices on either side (Fig. 2A). Although ATP was included in the purification buffer, ADP was found in the OSM‐3 nucleotide‐binding site (Fig. 2B), as is common with purified kinesins [11]. Accordingly, the region from Arg238 to Lys254, which encompasses the C‐terminal part of the Switch 2‐containing L11 loop and the N‐terminal part of the neighboring α4 helix, is disordered. This feature is shared by isolated ADP‐kinesins [32]. In addition, the L9 loop of ADP‐OSM‐3 is poorly ordered, with residues 195–201 unable to be built in the model, similar to, for example, the kinesin‐10 NOD [21] but in contrast to the helical conformation found in other ADP‐kinesin structures. As this loop comprises the Switch 1 motif and is part of the nucleotide‐binding site, this observation agrees with a lower involvement of Switch 1 in the binding of ADP [32] than of ATP [12]. ADP and the associated Mg^2+^ ion are however tightly bound to OSM‐3, as reflected by their mean temperature factors (about 41 and 44 Å^2^, respectively) that are similar to that of the nearby P‐loop region (residues 86–96; 39 Å^2^), taken as a reference, but lower than that of the complete model (52 Å^2^). Another, although indirect, indication of the tight binding of ADP is that OSM‐3 devoid of a nucleotide is unstable (see below).

Another characteristic of ADP‐OSM‐3 is that the neck linker is docked onto the core motor domain (Fig. 2A,C), the ‘docked’ state being a feature of microtubule‐bound ATP‐kinesins [27, 28, 29]. The docking of the neck linker requires a movement of the α4 helix relative to the core motor domain, and to the α6 helix in particular, to open up a hydrophobic pocket in which the first residue of the neck linker, a conserved isoleucine residue (Ile327 in OSM‐3), is accommodated [14, 20, 33]. In the case of ADP‐OSM‐3, the movement of α4 might be facilitated because the two loops (L11 and L12) flanking α4 are mobile in this structure (Fig. 2A). The docked neck linker is also stabilized by the cover neck bundle, a two‐stranded β‐sheet contributed by the N‐terminal end of the motor domain and by the neck linker [33, 34, 35]. In the case of OSM‐3, the additional β0 and β9 strands are constituted of only two residues (Figs 1A and 2D), therefore shorter than those seen in kinesin‐1. Such short β‐strands are a feature shared by the other kinesin‐2s whose motor domain structure has been determined [31, 36, 37]. The neck linker is further stabilized by a ‘latch’ formed by a conserved asparagine residue [35]. In OSM‐3, this residue (Asn334) interacts with Asn80 and Asn295 (Fig. 2D), similarly to what is observed in kinesin‐1. Asn80 is a kinesin conserved residue, whereas Asn295 is found in several kinesin families (e.g., kinesin‐1 and kinesin‐2) but not in all. As an example, a basic residue (Lys or Arg) is found in kinesin‐5 [38].

As expected, a structural comparison indicates that the structure of human kinesin‐1 closest to that of ADP‐OSM‐3 is the one of ADP‐kinesin‐1 with a docked neck linker (PDB id 1MKJ [33]; rmsd 1.07 Å on 289 Cαs superimposed; Fig. 2E, Table 2). To sum up, the ADP‐OSM‐3 motor domain shows a standard structure of ADP‐kinesins.

**Table 2:** Rmsd after superposition of OSM‐3 structures with those of human kinesin‐1. n.d., not determined

Kinesin‐1^a^	1BG2	1MKJ	4LNU	4HNA
ADP‐OSM‐3	1.49 Å (278 Cαs)^b^	1.07 Å (289 Cαs)	1.91 Å (271 Cαs)	1.37 Å (282 Cαs)
Nucleotide‐free OSM‐3 (Chain A)	n.d.	1.13 Å (296 Cαs)	1.93 Å (273 Cαs)	n.d.
AMPPNP‐OSM‐3 (Chain A)	n.d.	1.26 Å (298 Cαs)	2.12 Å (280 Cαs)	1.11 Å (319 Cαs)

^a^ Pdb id: 1BG2, ADP‐kinesin‐1 with a disordered neck linker [32]; 1MKJ, ADP‐kinesin‐1 with a docked neck linker [33]; 4LNU, tubulin‐bound nucleotide‐free kinesin‐1 [8]; 4HNA, tubulin‐bound ATP‐like‐kinesin‐1 [14] (see also Table 3)

^b^ Number of Cαs aligned. In the case of the comparison of AMPPNP‐OSM‐3 with the 4HNA structure, this number is substantially higher because the L9‐Switch 1 and L11‐Switch 2 regions are fully ordered in both structures hence are used in the superposition.

### A structure of nucleotide‐free OSM‐3

To obtain a structure of nucleotide‐free OSM‐3, we first treated the protein with apyrase, a protocol utilized for human kinesin‐1 [39]. However, OSM‐3 heavily precipitated in these conditions, showing that the stability in the absence of a nucleotide is kinesin‐dependent [40]. We reasoned that, by adding apyrase just before setting up crystallization trials, nucleotide‐free OSM‐3 might be incorporated and stabilized in a crystal before denaturing. Indeed, OSM‐3 crystals diffracting X‐rays up to a resolution of 2.3 Å were obtained in these conditions (Table 1). The structure was solved by molecular replacement taking that of ADP‐OSM‐3 as a search model and there are two virtually identical kinesin molecules per asymmetric unit (rmsd 0.44 Å; 308 Cα atoms aligned).

**Fig. 3:**
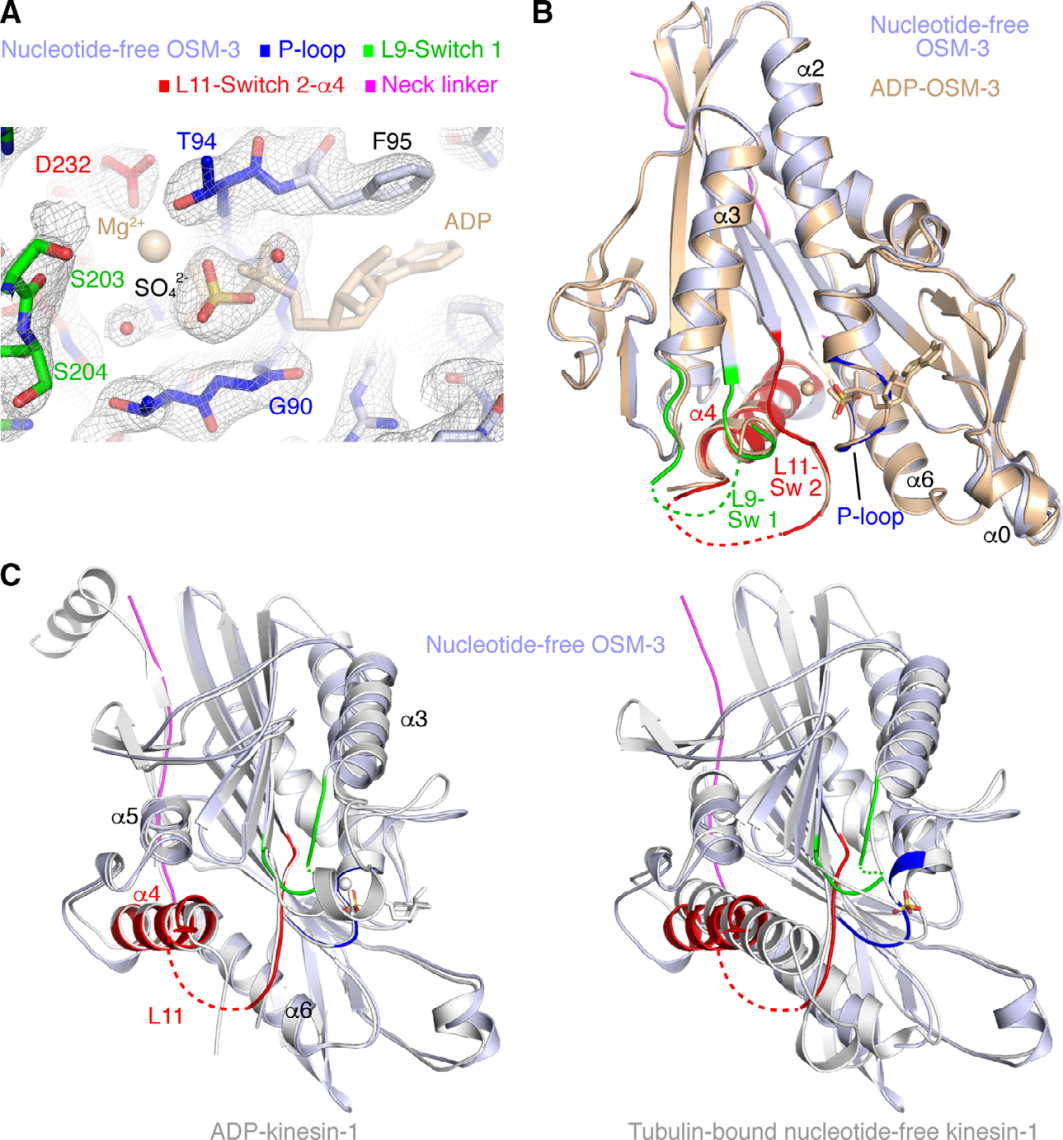
The structure of nucleotide‐free OSM‐3 (1–337 construct). Nucleotide‐free OSM‐3 is colored as indicated at the top of panel A. (A) The 2 *F*_obs _− *F*_calc_ electron density map (contoured at the 1 σ level) of the structure of apyrase‐treated OSM‐3. The Mg^2+^ ion and ADP from ADP‐OSM‐3 are shown in semi‐transparent wheat for reference. An electron density signal in the nucleotide‐binding site, modeled as a sulfate ion, overlaps with the ADP β phosphate. (B) Nucleotide‐free OSM‐3 adopts the same overall conformation than that of ADP‐OSM‐3 (wheat). (C) Comparison of nucleotide‐free OSM‐3 with ADP‐kinesin‐1 (left; pdb id 1MKJ) and tubulin‐bound nucleotide‐free kinesin‐1 (right; pdb id 4LNU). Kinesin‐1 is in gray.

The electron density maps indicated that the nucleotide‐binding site was indeed devoid of a nucleotide. Instead, an electron density was located close to the missing ADP β phosphate, as observed in other structures of nucleotide‐free kinesins [8, 39, 41, 42]. In the case of OSM‐3, this density is well accounted for by a sulfate ion that likely comes from the crystallization solution (Fig. 3A). The structure of nucleotide‐free OSM‐3 is however very similar to that of the ADP form (Fig. 3B), with an rmsd ranging from 0.49 (chain B) to 0.55 Å (chain A; 304 Cαs aligned in both cases). In particular, the L9 and L11 loops, belonging to the Switch 1 and Switch 2 regions, respectively, are disordered and the neck linker is docked onto the motor domain core. Relatedly, the structure of nucleotide‐free OSM‐3 is closer to that of ADP‐kinesin‐1 with a docked neck linker than to tubulin‐bound nucleotide‐free kinesin‐1 (Table 2, Fig. 3C). Our previous work on kinesin‐1 suggested that, in the absence of tubulin or microtubules, nucleotide‐free kinesin switches between ADP‐like and tubulin‐bound nucleotide‐free conformations [39]. In the OSM‐3 crystals, we did not capture this last conformation but that of ADP‐kinesin instead.

Although OSM‐3 devoid of a nucleotide is prone to precipitate, the approach we used, in which crystallization possibly happens just after nucleotide release, allowed us to obtain a nucleotide‐free structure of OSM‐3. We anticipate that this protocol will be useful for the structural study of other kinesins. It is similar to that used in the study of the kinesin‐3 KIF1A [43], where it led to ADP‐bound, Mg^2+^‐free kinesin rather than to a nucleotide‐free protein [44]. Finally, we note that the overall temperature factor of OSM‐3 was unexpectedly high (about 87 Å^2^) for a 2.3 Å resolution structure, likely reflecting further the instability of this kinesin in the absence of a nucleotide.

### The ATP‐like OSM‐3 structure

We also determined the structure of the OSM‐3 motor domain with bound AMPPNP. OSM‐3 was incubated with apyrase in the presence of this stable ATP analog and crystals diffracting X‐rays up to 1.9 Å were obtained (Table 1). The structure was solved by molecular replacement using ADP‐OSM‐3 as a search model, with two very similar molecules in the asymmetric unit (rmsd 0.50 Å; 335 Cαs compared).

**Fig. 4:**
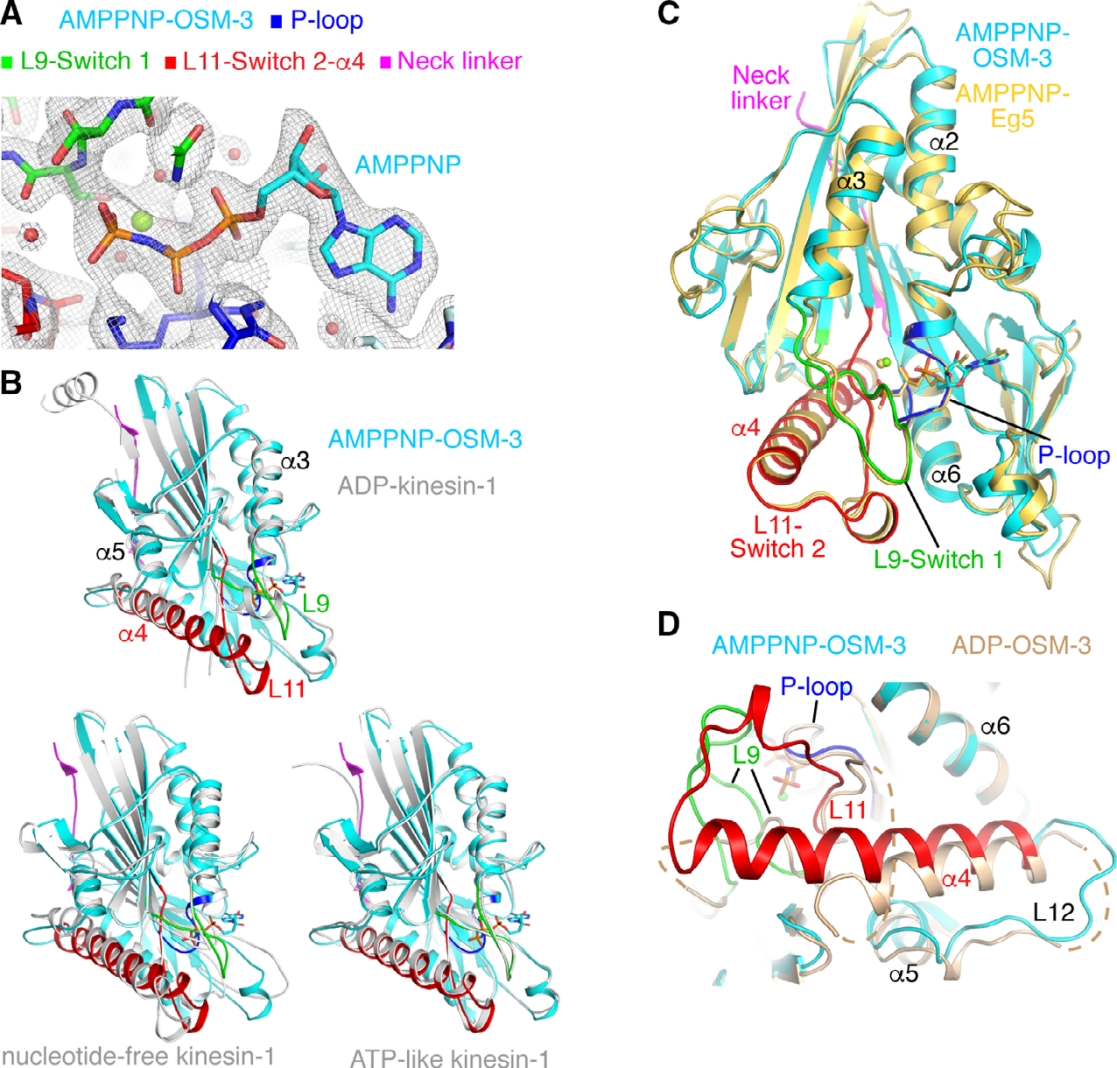
The structure of AMPPNP‐OSM‐3 (1–337 construct). AMPPNP‐OSM‐3 is colored as indicated in the top of panel A. (A) The 2 *F*_obs _− *F*_calc_ electron density map (contoured at the 1 σ level) of the nucleotide‐binding site of AMPPNP‐OSM‐3. (B) Superposition of kinesin‐1 structures (gray) with that of AMPPNP‐OSM‐3. The comparison is with ADP‐kinesin‐1 (top; pdb id 1MKJ), with tubulin‐bound nucleotide‐free kinesin‐1 (bottom, left; pdb id 4LNU), and with tubulin‐bound ATP‐like kinesin‐1 (bottom, right; pdb id 4HNA). Only the nucleotide and the associated Mg^2+^ ion from AMPPNP‐OSM‐3 are drawn. (C) Superposition of OSM‐3 with AMPPNP‐Eg5 (yellow; pdb id 3HQD). (D) Comparison of AMPPNP‐OSM‐3 with ADP‐OSM‐3 (wheat).

Electron density maps clearly indicate the presence of AMPPNP in the nucleotide‐binding site (Fig. 4A). Compared to kinesin‐1, AMPPNP‐OSM‐3 is closer to the tubulin‐bound ATP‐like structure (PDB id 4HNA) than to the nucleotide‐free form (PDB id 4LNU) or the ADP‐bound form (PDB id 1MKJ; Table 2, Fig. 4B). Actually, AMPPNP‐OSM‐3 has the same conformation as those of AMPPNP‐bound Eg5 (PDB code 3HQD; Fig. 4C) and KIF4 (PDB code 3ZFD) kinesins (rmsd 0.94 Å (321 Cαs compared) and 0.90 Å (311 Cαs compared), respectively). In particular, the Switch 2‐L11‐α4 region is fully ordered, with a short helical segment formed in L11 and α4 extended at its N‐terminal region by about 2.5 turns compared to ADP‐OSM‐3 (Fig. 4D). The L9‐Switch 1 region also becomes ordered and adopts a hairpin conformation. Therefore, the nucleotide‐binding site of AMPPNP‐OSM‐3 is in a ‘closed’ conformation. Interestingly, the superposition of the ADP‐ and AMPPNP‐OSM‐3 structures led to a rather high rmsd (1.36 Å, 268 Cαs compared). Much of this is due to a ~ 15° rotation of α4 and to a ~ 2 Å translation of α5 (Fig. 4D), two helices involved in the interaction with microtubules.

**Fig. 5:**
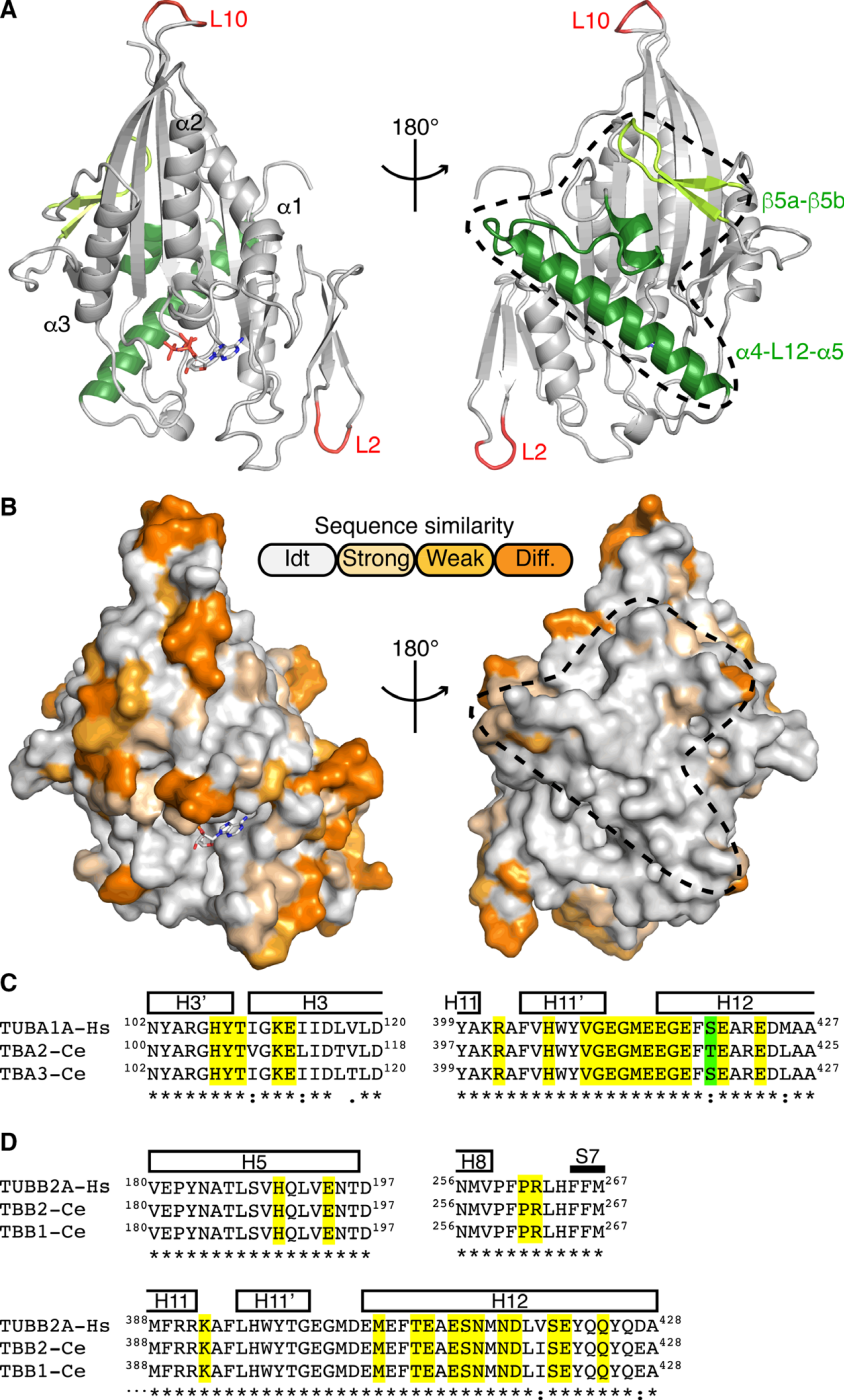
Human KIF17 motor domain homology model. (A) Representation of KIF17 with the two insertions compared to OSM‐3 indicated in red. Two ~ 180° views, centered either on the nucleotide‐binding site (left) or on the microtubule‐binding surface (delineated by a dashed line; right) are shown. The microtubule‐binding elements are colored in green. (B) Surface representation of KIF17 viewed along the same orientations as in the top panel and colored according to sequence conservation compared to OSM‐3, as indicated. (C, D) Sequence alignment centered on the kinesin‐interacting residues of human α‐tubulin (TUBA1A isoform, Uniprot Q71U36) with the two most abundant *Caenorhabditis elegans* α‐tubulin isoforms [56] (TBA2, Uniprot P34690, and TBA3, Uniprot P91910; panel C) and of human β‐tubulin (TUBB2A isoform, Uniprot Q13885) with the two most abundant *C. elegans* β‐tubulin isoforms [56] (TBB2, Uniprot P52275, and TBB1, Uniprot O17921; panel D). Tubulin residues that are < 5 Å distant from kinesin residues in the tubulin–kinesin‐1 complex [8, 14] are highlighted in yellow (identical residues) or in green (semi‐conserved residues).

We also modeled the structure of human KIF17 motor domain using that of AMPPNP‐OSM‐3 as a template. KIF17 has two insertions of three and four residues located in the L2 and L10 loops, respectively (Fig. 1A), at the distal ends of the motor domain (Fig. 5A). Structural analysis of the position of non‐conserved residues indicated that the microtubule‐binding surface is highly conserved as well as the residues of the nucleotide‐binding site (Fig. 5B). Moreover, the kinesin‐interacting residues of tubulin, as deduced from the structure of its complex with kinesin‐1 [8, 14], are conserved from *C. elegans* to human (Fig. 5C,D). Therefore, the conclusions reached from the OSM‐3 structure also likely apply to KIF17.

### A universal mechanism for ATP hydrolysis by kinesin proteins

**Fig. 6:**
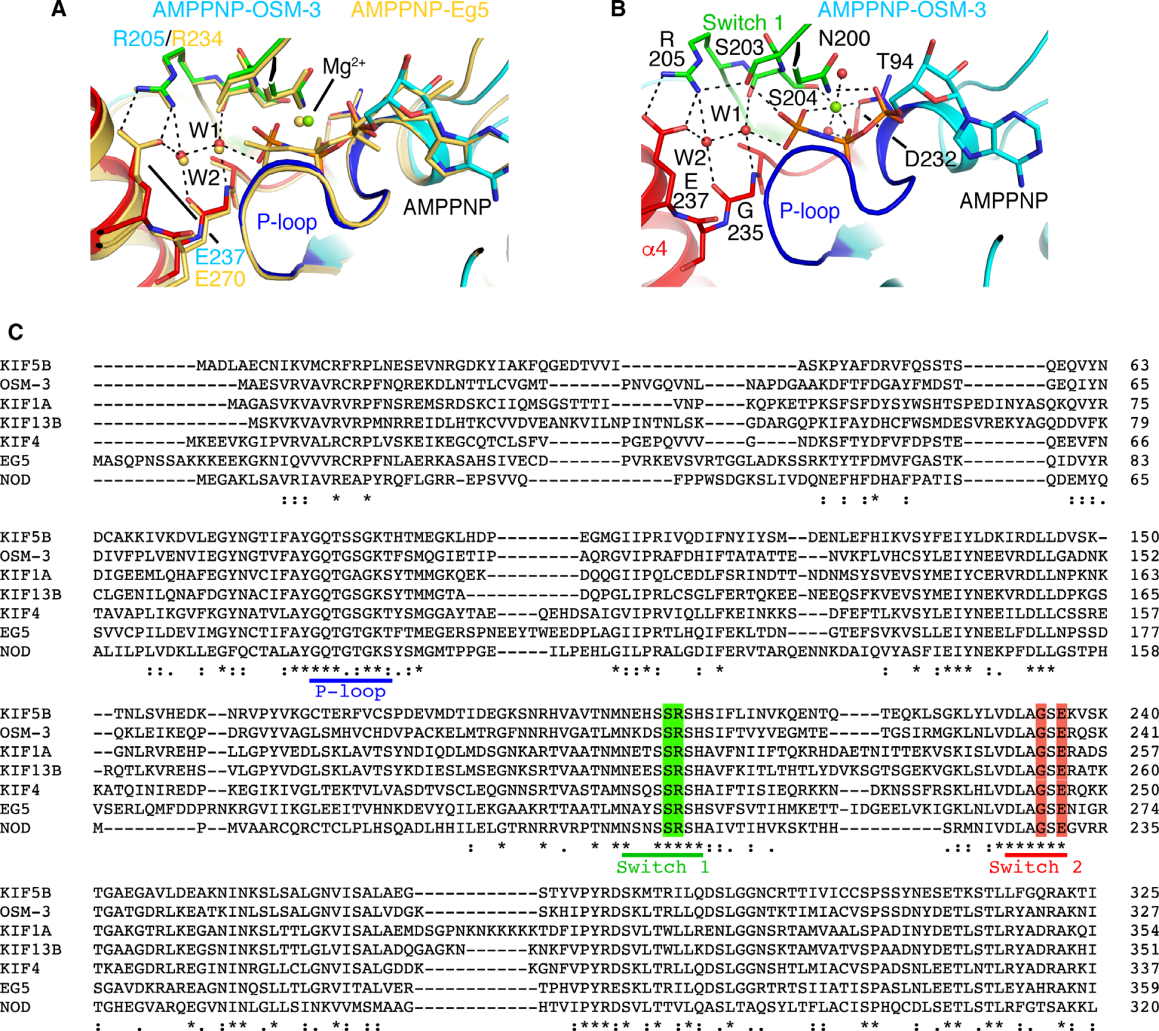
Structural basis for the ATP hydrolysis mechanism of OSM‐3. (A) Comparison of the nucleotide‐binding site of AMPPNP‐OSM‐3 with that of AMPPNP‐Eg5. The kinesins are colored as in Fig. 4. The two water molecules (W1 and W2) catalyzing the hydrolysis of ATP and defined in the Eg5 structure [12] are labeled. (B) Details of the AMPPNP‐OSM‐3 nucleotide‐binding site with key interactions shown as dashed lines. (C) Sequence alignment of the motor domain of kinesins whose crystal structures are compared in this manuscript. The residues directly interacting with W1 and W2 are highlighted in green (Switch 1 residues) or in red (Switch 2 residues). KIF5B, *Homo sapiens* kinesin‐1; OSM‐3, *Caenorhabditis elegans* kinesin‐2; KIF1A, *Mus musculus* kinesin‐3; KIF13B, *Rattus norvegicus* kinesin‐3; KIF4, *M. musculus* kinesin‐4; Eg5, *H*. *sapiens* kinesin‐5; NOD, *Drosophila melanogaster* kinesin‐10.

An ATPase mechanism involving two water molecules has been proposed from the structure of AMPPNP‐Eg5 [12], with a first water (named W1) positioned for γ‐phosphate attack and a second water (W2) acting as a general base. Because the structure of AMPPNP‐OSM‐3 is at a resolution that allows us to identify water molecules, we compared it more closely to that of ATP‐like Eg5. Strikingly, the nucleotide‐binding site is virtually identical, including many water molecules that are found at the same position in the vicinity of the nucleotide. In particular, two water molecules in the OSM‐3 nucleotide‐binding site overlap with W1 and W2 of Eg5 (Fig. 6A). The residues that are within hydrogen bond distance to these water molecules belong to the Switch 1 (Ser204 (carbonyl oxygen) and Arg205 in OSM‐3) and Switch 2 (Gly235 and Glu237) motifs (Fig. 6A,B). Interestingly, the Arg‐to‐Ala substitution in Switch 1 and those of Gly‐to‐Ala and Glu‐to‐Ala in Switch 2 lead to kinesin mutants having very low microtubule‐stimulated ATPase activities [27, 45].

Further comparisons indicate that, similar to isolated kinesin‐4 [13] and kinesin‐5 [12], AMPPNP‐OSM‐3 adopts the structure of tubulin‐bound or microtubule‐bound ATP‐like kinesin‐1 (Fig. 4B) [9, 14], kinesin‐6 [18], kinesin‐8 [19], and kinesin‐13 [15, 16, 17], suggesting that AMPPNP‐OSM‐3 displays the microtubule‐bound ATP conformation of OSM‐3. In contrast, such a conformation was not seen in isolated ATP‐like kinesin‐3s KIF1A [20] and KIF13B [22] or in kinesin‐10 NOD [21]. Several hypotheses can be put forth to explain this difference. First, it might reflect a structural feature specific to kinesin‐3 and kinesin‐10 motor domains. Another possibility is that, in the absence of tubulin or of microtubules, ATP‐like kinesins exist in equilibrium between different conformations. In some cases, the microtubule‐bound conformation can be trapped in the crystal, as seen for AMPPNP‐OSM‐3, whereas in other cases, as those of kinesin‐3 and kinesin‐10, the structure remains largely similar to what is observed in ADP‐kinesins. This second hypothesis seems more likely because the same ‘closed’ conformation of the nucleotide‐binding site in the ATP state is shared by kinesins as different as OSM‐3, a *C. elegans* motile kinesin‐2 with an N‐terminal motor domain, and mammalian non‐motile kinesin‐13s which depolymerize microtubules and have a central motor domain. In addition, the residues interacting with the W1 and W2 water molecules are fully conserved in kinesins (Fig. 6C) [6, 41]. Moreover, in the case of kinesin‐13s, the ‘closed’ conformation has been observed in complexes with tubulin [15, 16, 17] but not in the structure of AMPPNP‐ or ADP‐AlF_x_‐bound isolated motor domains [46, 47]. This proposal is reminiscent of the equilibrium between disordered and docked states adopted by the neck linker in the absence of microtubules [33], and of the switch between ADP‐like and tubulin‐bound nucleotide‐free‐like conformations of isolated nucleotide‐free kinesins [39].

To conclude, our work extends to the kinesin‐2 family the ATP hydrolysis mechanism already supported by structural data on kinesins of several families. Taken together, it suggests that this mechanism is universal within the kinesin superfamily.

## Experimental procedures

### Cloning, protein expression and purification

The plasmid containing the *C. elegans* OSM‐3 cDNA was a gift of R. Vale. To increase the probability of obtaining crystals, the genes of two fragments coding for the motor domain and either 10 (aa. 1–337) or 35 (aa. 1–362) additional C‐terminal residues were amplified by PCR. They were subcloned in a modified pET28 plasmid, introducing a sequence for an hexahistidine tag at the C terminus of the constructs, and an additional codon for an Ala residue just after the methionine initiation codon. Recombinant OSM‐3 constructs were overexpressed in *Escherichia coli* (BL21 CodonPlus) in LB medium after induction by 0.5 mm isopropyl β‐d‐1‐thiogalactopyranoside (IPTG) at 18 °C overnight. They were purified from the soluble fraction by Ni^2+^‐affinity chromatography (Histrap HP 5 ml; GE Healthcare, Vélizy, France) followed by gel filtration (Superdex 75 Increase 10/300 GL; GE Healthcare: Vélizy, France) in 25 mm Pipes‐K pH 6.8, 100 mm NaCl, 2 mm MgCl_2_, 1 mm EGTA, and 25 μm ATP. OSM‐3 proteins were concentrated to about 8 mg·mL^−1^, flash‐cooled in liquid nitrogen and stored at −80 °C until use.

### SEC‐MALLS analysis

Size‐exclusion chromatography (SEC) was carried out on a Prominence HPLC system (Shimadzu: Marne‐la‐Vallée, France) using a Superdex 200 Increase 10/300 GL (GE Healthcare) column in 25 mm Pipes‐K pH 6.8, 100 mm NaCl, 2 mm MgCl_2_, 1 mm EGTA, 25 μm ATP. Samples of 100 μL at about 2 mg·mL^−1^ OSM‐3 constructs were run at a 0.5 mL·min^−1^ flow rate. Detection was performed using a three‐detector static light‐scattering apparatus (MiniDAWN TREOS; Wyatt Technology, equipped with a quasi‐elastic light‐scattering module) and a refractometer (Optilab T‐rEX; Wyatt Technology, Toulouse, France). Calculations of the molecular weight were performed with the astra 6.1 software (Wyatt Technology) using a dn·dc^−1^ value of 0.183 mL·g^−1^.

### Interaction of OSM‐3 with microtubules

OSM‐3 was desalted using a Micro Bio‐spin 6 column (BioRad, Marnes‐La‐Coquette, France) to remove excess nucleotide. OSM‐3 (5 µm) was then incubated with 15 µm pig brain tubulin at 37 °C during 30 min in a microtubule‐assembly buffer (50 mm Mes‐K, pH 6.8, 30% glycerol, 0.5 mm EGTA, 6 mm MgCl_2_, 0.5 mm GTP), in the absence or in the presence of 2 mm ADP or 1 mm AMPPNP. After high speed centrifugation (300 000 ***g*** for 15 min at 35 °C), the supernatant and pellet were analyzed by SDS/PAGE stained with Coomassie blue.

In the case of the 1–337 construct, the identity of the kinesin band was confirmed by western blot (Fig. 1D). After SDS/PAGE separation, the proteins were transferred to a nitrocellulose membrane. The membrane was then blocked in 5% skimmed milk and incubated with a 1 : 2000 dilution of an anti‐His antibody (Sigma A7058, St. Quentin Fallavier, France). The blot was developed with a chemiluminescent substrate (Super Signal West Pico; Thermo, Les Ulis, France) and the proteins were visualized using a Fuji imager.

### Crystallization, structure determination, and refinement

#### ADP‐OSM‐3

Crystallization experiments were performed directly after protein purification. Crystals were obtained by vapor diffusion with the 1–362 construct in a solution consisting of 0.2 m KNO_3_ and 20% (W/V) polyethylene glycol 3350. They were harvested in the crystallization solution and flash‐cooled in liquid nitrogen. Data were collected at 100 K at the PROXIMA 2A beamline of the SOLEIL synchrotron. They were processed in the C2 space group with XDS [48] using the XDSME package [49]. The structure was solved by molecular replacement with PHASER [50] using the structure of the kinesin‐2 KIF3B (pdb id 3B6U [31]) as a search model. The structures were iteratively refined with BUSTER [51] with model building in Coot [52].

#### Nucleotide‐free OSM‐3

The incubation of OSM‐3 with apyrase led to protein precipitation, indicating that this kinesin is unstable in the absence of a nucleotide and of microtubules. To circumvent this limitation, we added 1 U·mL^−1^ apyrase (Sigma) to the ADP‐OSM‐3 solution just before setting up the crystallization experiments. Crystals were obtained with the 1–337 OSM‐3 construct in 20% (W/V) polyethylene glycol 3350, 170 mm (NH_4_)_2_SO_4_, and 0.1 m Mes buffer at pH 6.5. They were harvested in the crystallization solution supplemented with 18% glycerol then flash‐cooled in liquid nitrogen. X‐ray data were collected at the PROXIMA 1 beamline of the SOLEIL synchrotron and processed as described above in the case of ADP‐OSM‐3. The crystals belong to the P2_1_ space group. The structure was solved by molecular replacement using the ADP‐OSM‐3 structure as a search model and there are two molecules per asymmetric unit. It was refined as above.

#### AMPPNP‐OSM‐3

To load OSM‐3 with AMPPNP, the protein was incubated overnight at 4 °C with 1 U·mL^−1^ apyrase in presence of 4 mm AMPPNP. After a desalting step, 1 mm extra AMPPNP was added. Crystals of the 1–337 AMPPNP‐OSM‐3 construct were obtained in the same crystallization buffer as the one used for nucleotide‐free OSM‐3, but they belong to the C222_1_ space group. The same protocol was then used for data collection and processing, structure determination and refinement.

Data collection and refinement statistics are reported in Table 1. Figures of structural models were prepared with pymol (www.pymol.org).

### Structure comparison

Pairwise superposition of structures has been done with the ssm software [53] as implemented in Coot [52] without further optimization. Most structural comparisons were made with human kinesin‐1 (Table 2) because structures of this kinesin in different nucleotide states and either isolated or bound to tubulin or to microtubule are available. Further details on the kinesin structures used from structural comparisons are given in Table 3.

**Table 3:** Details of the kinesins used for structural comparisons. d, disordered; NF, nucleotide‐free; o, ordered.

	PDB	Reference	Tubulin‐bound	Nucleotide state	Neck linker conformation	L9‐Switch 1/L11‐Switch 2
Kinesin‐2						
OSM‐3	7A3Z	This study	no	ADP	Docked	d/d
OSM‐3	7A40	This study	no	NF	Docked	d/d
OSM‐3	7A5E	This study	no	AMPPNP	Docked	o/o
Kinesin‐1						
KIF5B	1BG2	[32]	no	ADP	Disordered	o/d
KIF5B	1MKJ	[33]	no	ADP	Docked	o/d
KIF5B	4LNU	[8]	yes	NF	Disordered^a^	d/o
KIF5B	4HNA	[14]	yes	ADP‐AlF_x_	Docked	o/o
Kinesin‐5						
Eg5	3HQD	[12]	no	AMPPNP	Docked	o/o
Kinesin‐4						
KIF4	3ZFD	[13]	no	AMPPNP	Docked	o/o

^a^ Whereas the kinesin construct used in the 4LNU structure was truncated after the first amino acid of the neck linker (Ile325 in human kinesin‐1), this peptide has been shown to be disordered in nucleotide‐free tubulin‐bound kinesin [27].

### Homology modeling of Human KIF17

The motor domain of human KIF17 (residues 1–346) was modeled using the I‐TASSER server with the ‘templates to guide I‐TASSER modeling’ option specifying the AMPPNP‐OSM‐3 structure at 1.9 Å resolution as template, but without sequence alignment [54]. The KIF17 model with the best estimated C‐score (1.90) and TM‐score (0.98 ± 0.05) was conserved. Superposition of the KIF17 model with the AMPPNP‐OSM‐3 crystal structure gives an rmsd ranging from 0.66 (chain B; 331 Cαs compared) to 0.72 Å (chain A; 333 Cαs compared).

## Conflict of interest

The authors declare no conflict of interest.

## Author contributions

KJV, JM, and BG conceived the study; PFV, JM, and BG planned experiments; PFV, MC, CV, JM, and BG performed experiments; PFV, JM, and BG analyzed data; KJV contributed reagents; and JM and BG wrote the manuscript with input from PFV and KJV.

## Data Availability

Coordinates and structure factors have been deposited with the Protein Data Bank with accession numbers 7A3Z (ADP‐OSM‐3), 7A40 (nucleotide‐free OSM‐3), and 7A5E (AMPPNP‐OSM‐3).
